# Phase 1b study of lenvatinib (E7080) in combination with temozolomide for treatment of advanced melanoma

**DOI:** 10.18632/oncotarget.5756

**Published:** 2015-10-15

**Authors:** David S. Hong, Razelle Kurzrock, Gerald S. Falchook, Corina Andresen, Jennifer Kwak, Min Ren, Lucy Xu, Goldy C. George, Kevin B. Kim, Ly M. Nguyen, James P. O'Brien, John Nemunaitis

**Affiliations:** ^1^ The University of Texas MD Anderson Cancer Center, Houston, TX, USA; ^2^ Sarah Cannon Research Institute at HealthONE, Denver, CO, USA; ^3^ Former employees of Eisai Inc., Woodcliff Lake, NJ, USA; ^4^ Eisai Inc., Oncology, Woodcliff Lake, NJ, USA; ^5^ Mary Crowley Cancer Research Center, Dallas, TX, USA

**Keywords:** lenvatinib, melanoma, pharmacodynamic, phase 1b, advanced solid tumors

## Abstract

**Objective and Methods:**

In this phase 1b study, patients with stage 4 or unresectable stage 3 melanoma were treated with escalating doses of lenvatinib (once daily) and temozolomide (TMZ) (days 1–5) in 28-day cycles, to determine the maximum tolerated dose (MTD) of the combination. Dose Level (DL)1: lenvatinib 20 mg, TMZ 100 mg/m^2^; DL2: lenvatinib 24 mg, TMZ 100 mg/m^2^; DL3: lenvatinib 24 mg, TMZ 150 mg/m^2^. Adverse events (AEs) were recorded and tumor response assessed per RECIST 1.0.

**Results:**

Dose-limiting toxicity occurred in 1 of 32 treated patients (DL1); MTD was not reached. The highest dose administered was lenvatinib 24 mg + TMZ 150 mg/m^2^. Most common treatment-related AEs included fatigue (56.3%), hypertension (53.1%), and proteinuria (46.9%). Overall objective response rate was 18.8% (6 patients), all partial response; (DL1, *n* = 1; DL3, *n* = 5). Stable disease (SD) ≥ 16 weeks was observed in 28.1% of patients (DL1 and DL2, *n* = 1 each; DL3, *n* = 7); 12.5% of patients had SD ≥ 23 weeks. Single and repeat-dose pharmacokinetics of lenvatinib were comparable across cycles and with concomitant TMZ administration.

**Conclusion:**

Lenvatinib 24 mg/day + TMZ 150 mg/m^2^/day (days 1–5) demonstrated modest clinical activity, an acceptable safety profile, and was administered without worsening of either lenvatinib- or TMZ-related toxicities in this patient group.

## INTRODUCTION

Melanoma has one of the fastest increasing incidence rates of any cancer [1, 2]. According to 2014 estimates, there were 76,100 new cases of, and 9710 deaths from, malignant melanoma in the United States. Approved treatments for metastatic melanoma have only recently demonstrated significant activity. These treatments include ipilimumab (human cytotoxic T-lymphocyte antigen 4-blocking antibody), vemurafenib, dabrafenib (*BRAF* inhibitors), trametinib (MEK inhibitor), pembrolizumab (anti-programmed cell death 1 antibody), and nivolumab (an anti-PD1 receptor immune checkpoint inhibitor monoclonal antibody). Median overall survival with standard treatment is 7–15.9 months, and response rates range from 10.2% to 53% [3–8]. Unfortunately, some patients do not respond, and most still develop recurrent disease.

Angiogenesis—the formation of new blood vessels—is critical for tumor survival and progression [1, 2]. Genetic aberrations associated with angiogenic signaling pathways mediated by growth factors, including vascular endothelial growth factor (VEGF), fibroblast growth factor (FGF), and platelet-derived growth factor (PDGF), have been correlated with progression in metastatic melanoma [1, 2].

Lenvatinib (E7080) is an orally active multikinase inhibitor of VEGF-receptor (VEGFR) 1–3, FGF-receptor (FGFR) 1–4, PDGF-receptor (PDGFR)-α, RET, and KIT proto-oncogenes [9]. In phase 1 studies, lenvatinib showed antitumor activity in solid-tumor patients at a maximum tolerated dose (MTD) of 25 mg/day [10, 11]. Temozolomide (TMZ) is an oral chemotherapeutic agent that shows evidence of response and similar efficacy to that of dacarbazine in melanoma, but it is also able to cross the blood–brain barrier, giving it a possible role in the treatment of melanoma patients with brain metastases [12]. TMZ is metabolized through nonenzymatic pH-dependent hydrolysis, and, therefore, has low potential for drug-drug interactions [13]. The combination of an alkylating agent and VEGF inhibitor has been previously proposed. In *in vitro* studies and *in vivo* xenograft models of melanoma, treatment with dacarbazine increased VEGF expression, providing a potential resistance mechanism to monotherapy [14, 15]. Preclinical data from a human melanoma xenograft study that evaluated a combination of lenvatinib and TMZ showed preliminary evidence of an additive efficacy (Eisai, Inc.; data on file).

We report here the phase 1b results of a phase 1/1b study conducted to determine the MTD and pharmacokinetic profile of lenvatinib when given once daily in combination with TMZ as treatment for advanced melanoma. The safety and tolerability of the combination, tumor response, and potential biomarkers of efficacy of this combination were also evaluated.

## RESULTS

### Patients

A total of 32 patients with metastatic melanoma were enrolled across the 3 dose levels (DLs): DL1, *n* = 6; DL2, *n* = 4; and DL3, *n* = 22. The demographics and baseline characteristics for the overall study population are summarized in Table 1. The median age of patients was 57.5 years (range, 24–81). The majority of patients (65.6%) had an Eastern Cooperative Oncology Group (ECOG) performance score of 1, 62.5% were male, and 84% had received at least 1 prior chemotherapy regimen. Two patients, both in DL3, had previously received ipilimumab. The *BRAF*^*V600E*^ tumor mutation was present in 7 (44%) of 16 evaluable patients and the *NRAS* tumor mutation was present in 6 (50%) of 12 evaluable patients.

**Table 1:** Baseline Patient Characteristics

Category	DL1 Lenvatinib 20 mg + TMZ 100 mg/m^2^ (*n* = 6)	DL2 Lenvatinib 24 mg + TMZ 100 mg/m^2^ (*n* = 4)	DL3 Lenvatinib 24 mg + TMZ 150 mg/m^2^ (*n* = 22)	Combined Total (*N* = 32)
**Age, years**				
Mean (SD)	62.5 (9.71)	57.0 (8.08)	53.2 (13.97)	55.4 (12.94)
Median	59.0	55.0	55.5	57.5
Range (Min, Max)	55, 81	50, 68	24, 79	24, 81
**Sex, *n* (%)**				
Male	5 (83.3)	3 (75.0)	12 (54.5)	20 (62.5)
Female	1 (16.7)	1 (25.0)	10 (45.5)	12 (37.5)
**Race, *n* (%)**				
Non-Hispanic White	4 (66.7)	4 (100)	19 (86.4)	27 (84.4)
Hispanic	1 (16.7)	0	3 (13.6)	4 (12.5)
African-American	1 (16.7)	0	0	1 (3.1)
**ECOG score^a^, *n* (%)**				
0	3 (50.0)	2 (50.0)	6 (27.3)	11 (34.4)
1	3 (50.0)	2 (50.0)	16 (72.7)	21 (65.6)
**Previous anticancer treatments, *n* (%)**				
Chemotherapy ≥ 1	5 (83.3)	3 (75.0)	19 (86.4)	27 (84.4)
Radiotherapy	3 (50.0)	3 (75.0)	9 (40.9)	15 (46.9)
Surgery	6 (100)	4 (100)	22 (100)	32 (100)
Other anticancer treatment regimens	3 (50.0)	2 (50.0)	13 (59.1)	18 (56.3)
**Previous chemotherapy regimens, *n* (%)**				
0	1 (16.7)	1 (25.0)	3 (13.6)	5 (15.6)
1	2 (33.3)	2 (50.0)	10 (45.5)	14 (43.8)
2	2 (33.3)	0	4 (18.2)	6 (18.8)
≥3	1 (16.7)	1 (25.0)	5 (22.7)	7 (21.9)
**Mutational status,^a^*n* (%)**				
*BRAF*	NA	NA	7 (43.8)	NA
*NRAS*	NA	NA	6 (50.0)	NA

a 16 And 12 patient tumors were evaluable for *BRAF* and *NRAS* testing, respectively.

DL, dose level; ECOG, Eastern Cooperative Oncology Group; NA, not available; SD, standard deviation; TMZ, temozolomide.

Overall, 22 (68.8%) patients discontinued the study due to progressive disease (PD) or clinical deterioration (DL1, *n* = 4; DL2, *n* = 3; DL3, *n* = 15). Additionally, 5 patients withdrew consent (DL1, *n* = 1; DL3, *n* = 4), and 1 patient from each group discontinued due to adverse events (AEs) AEs (DL1) and physician's decision (DL2). Three patients (9.4%) died either during study treatment or within 30 days after last dose (DL1, *n* = 2; DL3, *n* = 2).

### Dose-limiting toxicities and maximum tolerated dose

One dose-limiting toxicity (DLT) (grade 3 proteinuria) occurred in DL1. The MTD was not reached because no patients experienced a DLT at the highest combination dose (DL3). Investigators chose not to escalate the lenvatinib dosage beyond 24 mg/day (the established MTD for lenvatinib) or TMZ dose beyond 150 mg/m^2^ (the conventional dose level when combined with other drugs).

### Safety

The safety population was comprised of all 32 patients receiving at least 1 dose of study drug. Fatigue (56%), hypertension (53%), and proteinuria (47%) were the most commonly observed study-drug-related toxicities (Table 2). The most common National Cancer Institute Common Terminology Criteria for Adverse Events (version 3.0) grade 3 study-drug-related toxicities in the overall study population were asthenia (13%), hypertension (9%), fatigue (6%), hyponatremia (6%), and proteinuria (6%), with most grade 3 toxicities seen in DL3 (Table 2). Only 1 grade 4 drug-related toxicity (DL3; myocardial infarction) occurred and was handled through dose adjustment. Overall, 31.3% of patients experienced only grade 1–2 toxicities. A total of 3 patients died during treatment or within 30 days of last dose. All 3 deaths appear to have been due to disease progression.

**Table 2:** Adverse Events Occurring in ≥ 20% of Overall Patients and CTC Grade 3 Drug-related Adverse Events Occurring in at Least 2 Patients

	DL1 Lenvatinib 20 mg + TMZ 100 mg/m^2^ (*n* = 6) *n* (%)	DL2 Lenvatinib 24 mg + TMZ 100 mg/m^2^ (*n* = 4) *n* (%)	DL3 Lenvatinib 24 mg + TMZ 150 mg/m^2^ (*n* = 22) *n* (%)	Combined Total (*N* = 32) *n* (%)
**Patients with treatment-related all-grade AEs**	6 (100)	4 (100)	20 (90.9)	30 (93.8)
**≥20% All-grade drug-related TEAEs**				
Fatigue	4 (66.7)	3 (75.0)	11 (50.0)	18 (56.3)
Hypertension	5 (83.3)	1 (25.0)	11 (50.0)	17 (53.1)
Proteinuria	3 (50.0)	1 (25.0)	11 (50.0)	15 (46.9)
Hypothyroidism	3 (50.0)	1 (25.0)	10 (45.5)	14 (43.8)
Anorexia	3 (50.0)	2 (50.0)	9 (40.9)	14 (43.8)
Nausea	2 (33.3)	1 (25.0)	9 (40.9)	12 (37.5)
Vomiting	2 (33.3)	0	10 (45.5)	12 (37.5)
Diarrhea	2 (33.3)	1 (25.0)	8 (36.4)	11 (34.4)
Thrombocytopenia	0	0	8 (36.4)	8 (25.0)
Asthenia	1 (16.7)	1 (25.0)	6 (27.3)	8 (25.0)
Blood thyroid stimulating hormone increased	0	1 (25.0)	6 (27.3)	7 (21.9)
**Patients with CTC grade 3 drug-related TEAEs^a^**	2 (33.3)	2 (50.0)	9 (40.9)	13 (40.6)
CTC grade 3 drug-related TEAEs				
Asthenia	0	1 (25.0)	3 (13.6)	4 (12.5)
Hypertension	1 (16.7)	0	2 (9.1)	3 (9.4)
Fatigue	0	0	2 (9.1)	2 (6.3)
Hyponatremia	0	0	2 (9.1)	2 (6.3)
Proteinuria	1 (16.7)	1 (25.0)	0	2 (6.3)

a Grade 4 drug-related TEAE occurred in 1 patient (myocardial infarction).

AEs, adverse events; CTC, common terminology criteria; DL, dose level; TEAE, treatment-emergent adverse event; TMZ, temozolomide.

### Pharmacokinetics

Overall, single-dose and repeat-dose PK parameters of lenvatinib were comparable across cycles and with concomitant TMZ administration (Supplementary Table 1). Lenvatinib area under the concentration-time curve extrapolation to time (AUC_(0–τ)),_ area under the curve from time 0 extrapolated to infinite time (AUC_(0–inf)_), and peak plasma concentration (C_max_) were similar across the 3 cohorts following single- and repeat-dose administration. Similarly, the median time to peak concentration (t_max_), terminal 1/2 life (t_1/2)_, and clearance after oral administration (CL/F) estimates for lenvatinib were independent of the dose of lenvatinib or TMZ regimen. Following administration of treatment on cycle 2 day 1, the median t_max_ ranged between 1 to 8 hours across the cohorts, and there was no apparent accumulation upon repeated administration of lenvatinib alone or in combination with TMZ (data not shown).

### Tumor response and duration of treatment

Patients in DL1 received a median of 3.0 (range 1.5) cycles of both lenvatinib and TMZ, patients in DL2 received a median of 3.0 (2.0, 4.0) cycles of lenvatinib and 2.5 (2.0, 4.0) cycles of TMZ, and patients in DL3 received a median of 4.0 (1.0, 7.0) cycles of lenvatinib and 3.5 (1.0, 7.0) cycles of TMZ. At the time of data cutoff, there were 4 partial responses (PRs) observed, with 3 patients still undergoing treatment; in an updated analysis, 2 out of those 3 patients additionally achieved PR (personal communication, D.S. Hong). A best response of PR was, therefore, observed in 6/32 (18.8%) patients, with 1 PR (16.7%) in DL1 and 5 PRs in DL3 (22.7%; Table 3). All 6 patients who achieved PR received lenvatinib for at least 3 treatment cycles (range, 3–11 cycles). Stable disease (SD) ≥ 16 weeks was achieved by 9/32 (28.1%) patients; 1 patient each in DL1 and DL2, and 7 (31.8%) patients in DL3. Four (18.2%) patients in DL3 had durable SD. Median progression-free survival (PFS) in DL3 was 5.4 months.

**Table 3:** Best Overall Tumor Responses

Best Overall Tumor Response^a^	DL1 Lenvatinib 20 mg + TMZ 100 mg/m^2^ (*n* = 6) *n* (%)	DL2 Lenvatinib 24 mg + TMZ 100 mg/m^2^ (*n* = 4) *n* (%)	DL3 Lenvatinib 24 mg + TMZ 150 mg/m^2^ (*n* = 22) *n* (%)	Combined Total (*N* = 32) *n* (%)
Complete response	0	0	0	0
Partial response	1 (16.7)	0	5 (22.7)	6 (18.8)
Stable disease	4 (66.7)	2 (50.0)	9 (40.9)	15 (46.9)
≥16 Weeks	1 (16.7)	1 (16.7)	7 (31.8)	9 (28.1)
≥23 Weeks^b^	0	0	4 (18.2)	4 (12.5)
Progressive disease	1 (16.7)	1 (25.0)	5 (22.7)	7 (21.9)
Unknown^c^	0	1 (25.0)	3 (13.6)	4 (12.5)

DL, dose level; SD, stable disease; TMZ, temozolomide.

a Responses evaluated based on RECIST 1.0 (Response Evaluation Criteria In Solid Tumors version 1.0). Responses for 3 patients were updated after the primary analysis because they were still on treatment at data cut-off: 2 Patients achieved partial responses, 1 patient achieved stable disease ≥23 weeks (personal communication, D.S. Hong).

b Durable SD is defined as SD lasting ≥23 weeks.

c Unknown – not assessable or insufficient data.

**Figure 1:**
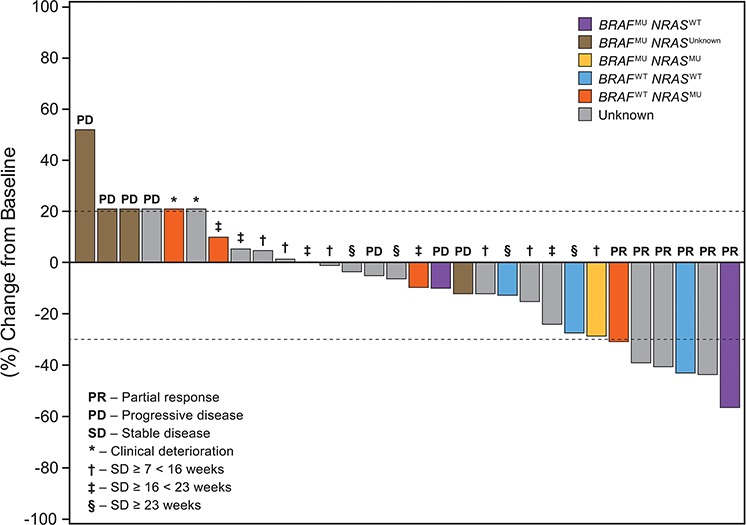
Patients with advanced/metastatic melanoma treated with the combination of lenvatinib and temozolomide *3 Patients with PD and 2 patients with clinical deterioration were included in the waterfall arbitrarily at a 21% increase. Responses for 3 patients were updated after the primary analysis, since they were still on treatment at data cut-off: 2 Patients achieved partial responses, 1 patient achieved stable disease ≥23 weeks (personal communication, D.S. Hong).

Figure 1 represents the maximum percentage change of target lesions from baseline in patients from DL3 stratified by *BRAF* and *NRAS* mutation status. Patients with wild type *BRAF* (BRAFWT) status (*n* = 7) seemed to have better overall response on treatment (*P* = 0.007). Two *BRAF*^WT^ patients achieved PR; 5 had SD ≥ 16 weeks (2 had durable [ie, > = 23 weeks] SD). There was no significant correlation between *NRAS* mutation status and response (*n* = 5; *P* = 0.69).

## DISCUSSION

The MTD was not reached in this phase 1b combination treatment portion of the study because no patients experienced a DLT at the maximum administered dose of the combination treatment (DL3 once daily); however, the investigators felt it would have been inappropriate to further escalate either one of the drugs. The single DLT reported in this population was grade 3 proteinuria in DL1. The highest dose for further clinical evaluation of this combination was defined as lenvatinib 24 mg/day, days 1–28, plus TMZ 150 mg/m^2^, days 1–5.

The combination therapy of lenvatinib and TMZ generally had an acceptable toxicity profile in patients with advanced melanoma, and it was given at the highest level of DL3 without apparent worsening of either lenvatinib- or TMZ-related toxicities. The most common treatment-emergent adverse events (TEAEs) considered related to study drug by investigators included fatigue, hypertension, proteinuria, hypothyroidism, and anorexia. The incidences of several TEAEs, including hypothyroidism and thrombocytopenia (46.9% each), in this combination study were higher compared with those in a study of lenvatinib alone (15.6% and 13.0%, respectively). This difference may be due to the combination treatment (lenvatinib plus TMZ) or due to TMZ alone.

TMZ has a safety and efficacy profile similar to the current standard of care, dacarbazine. A benefit of adding TMZ to metastatic melanoma therapy may be its ability to cross the blood-brain barrier [12]. Single-agent TMZ has been associated with response rates of 0% to 29% in patients with metastatic melanoma [12]. In combination with interferon alpha, response rates of 13% to 23% have been reported [12]. An uncontrolled phase 2 study of TMZ in combination with sorafenib also demonstrated activity in TMZ-naïve patients with melanoma, including patients with brain metastases [16].

In the present study of TMZ and lenvatinib as a combination therapy, we observed a best overall response of PR in 18.8%, and SD ≥ 16 weeks in 28.1% of the overall population. In addition, 4 patients in DL3 experienced durable SD ≥ 23 weeks. Best overall response was not evaluable or censored in 4 patients. However, the response rates observed with TMZ and lenvatinib in the present study are lower than a 61% objective response rate (44 of 72 patients) observed in patients with melanoma receiving a combination of ipilimumab and nivolumab [17], and lower than a 31.7% objective response rate (38 or 120 patients) observed in a phase 3 trial of nivolumab in patients with metastatic melanoma whose disease had progressed after previous treatment with ipilimumab, or treatment with ipilimumab and a *BRAF* inhibitor if their tumors harbored a *BRAF* V600E mutation [18].

Overall, in our analysis of pharmacokinetic parameters, we found that the single-dose and repeat-dose pharmacokinetics of lenvatinib were comparable across cycles and with concomitant TMZ administration. Estimates of t_1/2,_ t_max_, and CL/F for lenvatinib were independent of the dose of lenvatinib or TMZ. The pharmacokinetics of lenvatinib 24 mg in combination with TMZ was comparable to that seen when lenvatinib was given as a monotherapy and there was no evidence for accumulation upon repeated administration of lenvatinib alone or in combination with TMZ.

Angiopoietins are ligands of the endothelial cell receptor soluble Tie-2 (sTie-2) and have crucial roles in tumor angiogenic switches [1]. In malignant melanoma, soluble angiopoietin-2 (sAng-2) levels are elevated and may function as part of an autocrine Ang-2/Tie2 growth loop. Circulating levels of sAng-2 have been correlated with tumor load, disease stage, and overall survival [19]. The relationship observed between maximum tumor shrinkage (MTS) and change in levels of Ang-2 in the current study suggests that this angiogenic pathway may be associated with the activity of lenvatinib.

The tumor gene mutation analyses performed in this study indicated that *BRAF_WT_* status appeared to correlate with better response to this combination treatment. No clear correlation between response and *NRAS* mutation status could be made. Due to the fairly small sample size and exploratory nature of these biomarker analyses, these findings should be further explored in larger ongoing studies of lenvatinib.

In conclusion, the combination of lenvatinib with TMZ had an acceptable toxicity profile and can be administered without increase in toxicity of either agent. This combination therapy demonstrated modest clinical activity in a population of patients with unresectable stage 3 or stage 4 melanoma. These results will be further confirmed in ongoing phase 2 studies of lenvatinib in advanced melanoma. Additional studies are also underway to further explore the potential role of lenvatinib in treatment of advanced or metastatic melanoma.

## MATERIALS AND METHODS

### Patients

This was a phase 1/1b, open-label study (NCT00121680) conducted at the University of Texas MD Anderson Cancer Center and the Mary Crowley Cancer Research Center. The main inclusion criteria were: patients aged ≥ 18 years with a histological and/or cytological confirmed diagnosis of advanced or metastatic melanoma untreatable by standard therapies; melanoma lesions amenable to biopsy; adequate renal, hepatic, and hematologic parameters; and an ECOG performance status of 0 or 1. Patients who were intolerant of TMZ or any of its excipients, pregnant, required full-dose aspirin or chronic nonsteroidal anti-inflammatory drugs, or were untreated for or had unstable central nervous system metastases were excluded.

The study was conducted in accordance with World Medical Association Declaration of Helsinki and the International Conference on Harmonization of Technical Requirements for Registration of Pharmaceuticals for Human Use guidelines. Prior to study participation, all participants provided written informed consent. The Mary Crowley Cancer Research Center and University of Texas MD Anderson Cancer Center Institutional Review Boards approved the study. A separate publication details results of the monotherapy portion of the phase 1 study of lenvatinib in solid tumors (including a melanoma population).

### Study design

This phase 1b study utilized a standard “3+3” dose-escalation scheme to evaluate the MTD of lenvatinib in combination with TMZ. Patients were treated in sequential cohorts of escalating dosing of lenvatinib (continuous once daily) and TMZ (days 1–5) in a 28-day treatment cycle. The initial doses were lenvatinib 20 mg once daily with TMZ 100 mg/m^2^/day for days 1–5 (DL1). Subsequent doses were lenvatinib 24 mg with TMZ 100 mg/m^2^ (DL2) and lenvatinib 24 mg with TMZ 150 mg/m^2^ (DL3).

DLTs were assessed once the first 3 patients completed 1 treatment cycle. Any of the following events was defined as a DLT: any hematologic toxicity of grade 3 or higher; any nonhematologic toxicity of grade 3 or higher, with the exception of grade 3 hypertension that could be controlled (via intensification of single agent antihypertensive treatment or by adding a second antihypertensive); and any failure to administer ≥ 75% of the study drugs during cycle 1 due to treatment-related toxicities. The MTD was defined as the highest dose tolerated by a minimum of 5 out of the first 6 treated patients.

### Procedures

Blood samples were collected on cycle 1, day 1 (C1D1) immediately prior to the first dose of lenvatinib and at 0.25, 0.5, 1, 1.5, 2, 2.5, 3, 5, 8, and 24 hours following administration on C1D1 and day 1 cycle 2. Additional samples were collected prior to lenvatinib administration on days 8, 15, and 22 of cycle 1, as well as on day 1 of cycles 3 and 4. T_max_, C_max_, C_trough_, AUC_0-t_, CL (total body clearance from plasma), Vss (volume of distribution at steady rate), and t_1/2_ were evaluated.

Pharmacodynamic assessments of lenvatinib were conducted based on serum samples collected prior to study drug administration and at 2 hours following administration on C1D1, as well as prior to administration on days 8, 15, and 22 of cycle 1. Serum samples were tested for angiogenesis-related markers (MDS Pharma/Clearstone Central Lab) and apoptosis-related (Pathway Diagnostics/Quest) markers. These angiogenesis-related markers were PDGF-homodimer BB, sTie-2 (receptor expressed by endothelial cells), angiopoietin-1 (Tie-2 ligand), soluble E-selectin (mediates leukocyte and tumor cell rolling), and soluble c-kit. The apoptosis-related markers assessed were cytochrome C (a measure of intrinsic apoptotic pathway activation) and M30 neoantigen (caspase-cleaved cytokeratin-18, also a terminal apoptotic product for epithelial-derived tumors). sTie-2, angiopoietin-1, PDGF-BB, soluble e-selectin, soluble c-kit, cytochrome C, and M30 were measured by enzyme-linked immunosorbent assay. PDGF-BB was assayed by Luminex Technology using Growth Factor Buffer Reagent and a Human Custom Multiplex Antibody Bead Kit from BioSource Invitrogen (Frederick, MD). Pretreatment (baseline) and posttreatment changes in levels of additional serum cytokine and angiogenic factors (CAF), including Ang-2, sVEGFR1-3, sTie-2, PDGF-AB, PDGF-BB, VEGFA, FGF4, VEGFD, hepatocyte growth factor, FMS-related tyrosine kinase 3 ligand (FLT3LG), and placental growth factor were evaluated. CAFs were measured in house by Eisai.

Safety assessments were performed in each cycle by physical examination, weekly blood pressure monitoring, periodic measurement of vital signs and electrocardiograms, ECOG performance status evaluation, and regular monitoring of hematology, blood chemistry, and urine values. All AEs and serious AEs were recorded.

Tumor assessments were conducted at baseline and approximately every 8 weeks during treatment. Responses were confirmed after ≥ 30 days using Response Evaluation Criteria in Solid Tumors (RECIST), version 1.0 [20]. Tumor responses were assessed by computed tomography, magnetic resonance imaging, or, in the case of skin lesions, photography and clinical examination. Tumor responses were classified as either complete response (CR), PR, SD, or progressive disease (PD). SD was further stratified as SD maintained for ≥ 7weeks and ≥ 16 weeks, and durable SD was defined as SD maintained for ≥ 23 weeks.

### Tumor mutation analysis

Paraffin-embedded tumor samples were macrodissected using a dissecting microscope and analyzed for *BRAF* and *NRAS* mutations at the Clinical Laboratory Improvement Amendments-certified Molecular Diagnostics Laboratory at MD Anderson using standard operating procedures.

### Statistical analysis

Baseline and demographic variables including age, gender, race, height, weight, and ECOG performance score were summarized using descriptive statistics. Plasma concentrations of lenvatinib were analyzed and summarized using descriptive statistics for the following pharmacokinetic parameters: CL/F, AUC, C_max_, C_trough_, t_max_, and t_1/2_. Pearson and Spearman correlation coefficients were calculated between pharmacokinetic parameters and serum biomarkers at different time points compared with baseline to examine the correlation between drug exposure and biomarkers. Univariate and multivariate Cox proportional hazards models and log-rank tests were used to examine the relationship between PFS and baseline (and change from baseline) concentrations in angiogenesis and apoptosis biomarkers. Cumulative chi-square test was used to examine the correlation between mutation/serum biomarkers and best overall response. Pearson and Spearman correlation coefficients were also calculated between percent MTS (defined as percent change in sum of longest diameter from baseline to nadir using RECIST criteria) and serum biomarkers at different time points compared with baseline.

## SUPPLEMENTARY DATA



## References

[1] Emmett MS, Dewing D, Pritchard-Jones RO (2011). Angiogenesis and melanoma—from basic science to clinical trials. American Journal of Cancer Research.

[2] Ria R, Reale A, Castrovilli A (2010). Angiogenesis and progression in human melanoma. Dermatology Research and Practice.

[3] Chapman PB, Einhorn LH, Meyers ML (1999). Phase III multicenter randomized trial of the Dartmouth regimen versus dacarbazine in patients with metastatic melanoma. Journal of Clinical Oncology.

[4] Sosman JA, Kim KB, Schuchter L (2012). Survival in BRAF V600-mutant advanced melanoma treated with vemurafenib. The New England Journal of Medicine.

[5] Hodi FS, O'Day SJ, McDermott DF (2010). Improved survival with ipilimumab in patients with metastatic melanoma. The New England Journal of Medicine.

[6] Hauschild A, Grob JJ, Demidov LV (2012). Dabrafenib in BRAF-mutated metastatic melanoma: a multicentre, open-label, phase 3 randomised controlled trial. Lancet.

[7] Flaherty KT, Robert C, Hersey P (2012). Improved survival with MEK inhibition in BRAF-mutated melanoma. The New England Journal of Medicine.

[8] Ribas A, Hodi FS, Kefford R (2014). Efficacy and safety of the anti-PD-1 monoclonal antibody MK-3475 in 411 patients (pts) with melanoma (MEL) [abstract]. Journal of Clinical Oncology.

[9] Matsui J, Funahashi Y, Uenaka T, Watanabe T, Tsuruoka A, Asada M (2008). Multi-kinase inhibitor E7080 suppresses lymph node and lung metastases of human mammary breast tumor MDA-MB-231 via inhibition of vascular endothelial growth factor-receptor (VEGF-R) 2 and VEGF-R3 kinase. Clinical Cancer Research.

[10] Yamada K, Yamamoto N, Yamada Y (2011). Phase I dose-escalation study and biomarker analysis of E7080 in patients with advanced solid tumors. Clinical Cancer Research.

[11] Boss DS, Glen H, Beijnen JH (2012). A phase I study of E7080, a multitargeted tyrosine kinase inhibitor, in patients with advanced solid tumours. British Journal of Cancer.

[12] Quirt I, Verma S, Petrella T, Bak K, Charette M (2007). Temozolomide for the treatment of metastatic melanoma: a systematic review. The Oncologist.

[13] Baker SD, Wirth M, Statkevich P (1999). Absorption, metabolism, and excretion of 14C-temozolomide following oral administration to patients with advanced cancer. Clinical Cancer Research.

[14] Lev DC, Ruiz M, Mills L, McGary EC, Price JE, Bar-Eli M (2003). Dacarbazine causes transcriptional up-regulation of interleukin 8 and vascular endothelial growth factor in melanoma cells: a possible escape mechanism from chemotherapy. Molecular Cancer Therapeutics.

[15] Lev DC, Onn A, Melinkova VO (2004). Exposure of melanoma cells to dacarbazine results in enhanced tumor growth and metastasis *in vivo*. Journal of Clinical Oncology.

[16] Amaravadi RK, Schuchter LM, McDermott DF (2009). Phase II trial of temozolomide and sorafenib in advanced melanoma patients with or without brain metastases. Clinical Cancer Research.

[17] Postow MA, Chesney J, Pavlick AC (2015). Nivolumab and ipilimumab versus ipilimumab in untreated melanoma. The New England Journal of Medicine.

[18] Weber JS, D'Angelo SP, Minor D (2015). Nivolumab versus chemotherapy in patients with advanced melanoma who progressed after anti-CTLA-4 treatment (CheckMate 037): a randomised, controlled, open-label, phase 3 trial. The Lancet Oncology.

[19] Helfrich I, Edler L, Sucker A (2009). Angiopoietin-2 levels are associated with disease progression in metastatic malignant melanoma. Clinical Cancer Research.

[20] Therasse P, Arbuck SG, Eisenhauer EA (2000). New guidelines to evaluate the response to treatment in solid tumors. European Organization for Research and Treatment of Cancer, National Cancer Institute of the United States, National Cancer Institute of Canada. Journal of the National Cancer Institute.

