# Supplementary material for: Phase 1b study of lenvatinib (E7080) in combination with temozolomide for treatment of advanced melanoma
Source: Oncotarget. 2015 Oct 15;6(40):43127–34. doi: 10.18632/oncotarget.5756 (PMC4767496; doi:10.18632/oncotarget.5756)
Supplement: Supplementary file 1 [file oncotarget-06-43127-s001.pdf]

SUPPLEMENTARY DATA

Pharmacodynamics

Among the various pretreatment (baseline) and posttreatment serum-circulating factors that were tested, lower ratio of angiopoietin-2 on cycle 1 day 22 to baseline (C1D1) showed an association with greater tumor shrinkage (Spearman's correlation r = 0.66; $P = 0.014$) (Supplemental Figure 1, in Dose Level 3 [DL3] patients). The ratio of soluble Tie2 receptor (sTie-2) levels at 2 hours postdose to C1D1 was negatively associated with tumor shrinkage (Spearman's correlation r = −0.55; $P = 0.018$). Additionally, increase in proto-oncogene c-kit levels at 2 hours postdose on C1D1 ($n = 22$) and on cycle 1 day 15 (C1D15) ($n = 17$), as well as decrease in sTie-2 levels on C1D15 after treatment ($n = 17$), were also associated with longer progression-free survival following treatment ($P = 0.04$, $P = 0.02$, and $P = 0.04$, respectively).

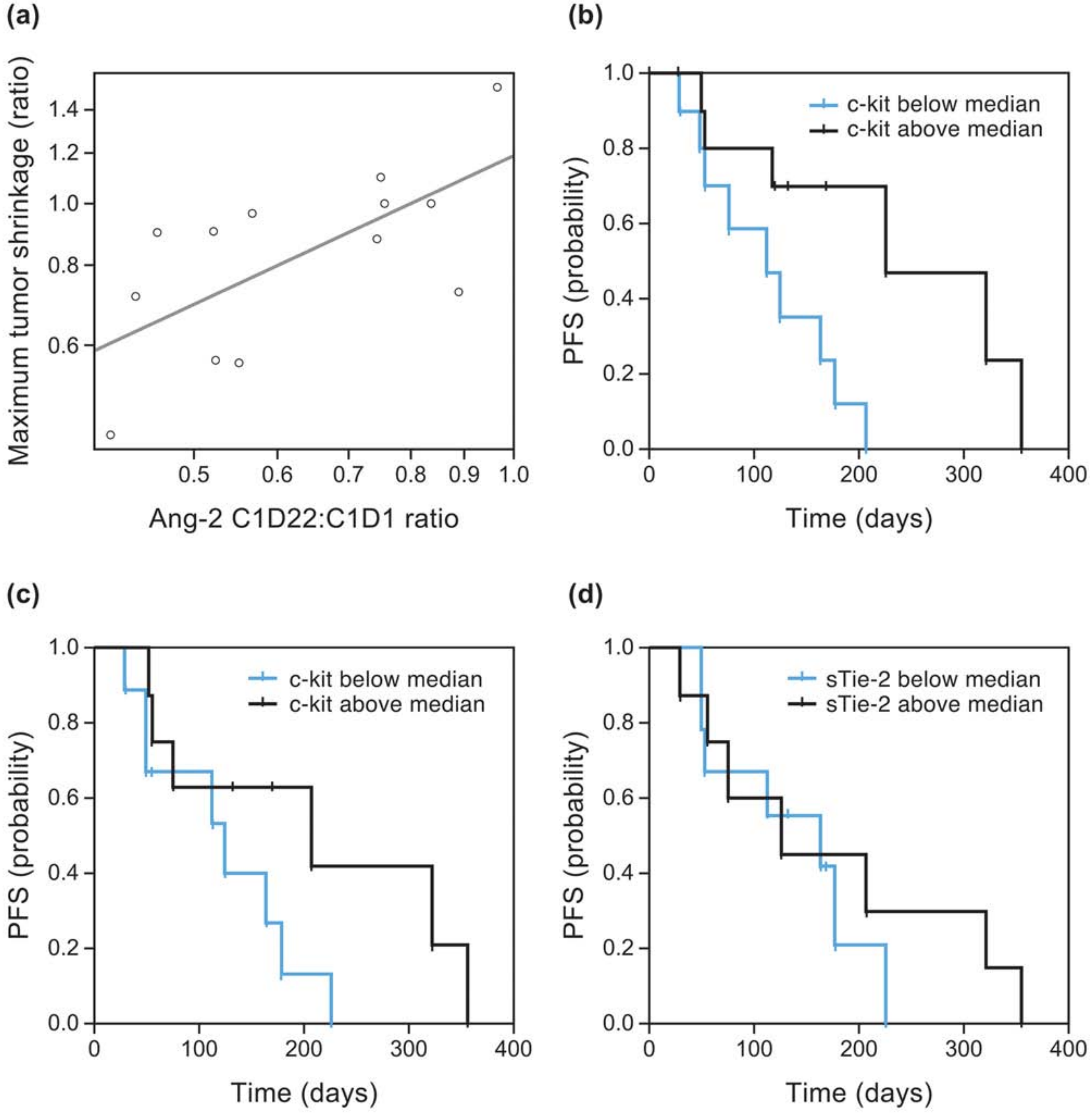

Ang-2, angiopoetin-2; C1D1, Cycle 1 Day 1; C1D22, Cycle 1 Day 22; DL, dose level; PFS, progression-free survival; RECIST, Response Evaluation Criteria in Solid Tumours; TMZ, temozolomide.

Supplementary Figure S1: a. Correlations of changes in levels of serum cytokines and angiogenic factors with clinical outcome in patients in DL3 (lenvatinib 24 mg + TMZ 150 mg/m^2). Ang-2 ratio (Cycle 1 Day 22 to Cycle 1 Day 1 [baseline]) versus maximum tumor shrinkage (tumor size at best tumor shrinkage/tumor size at pretreatment, measured per RECIST 1.0) (n = 13). Kaplan-Meier survival curves of progression-free survival for patients below and above the median change from baseline for c-kit **b.** 2 hours after treatment (n = 22) and **c.** on cycle 1 day 15 (n = 17). d. Kapan-Meier survival curve of progression-free survival for patients below and above the median change from baseline for sTie-2 on cycle 1 day 15 (n = 17).

Supplementary Table S1: Summary of Pharmacokinetic Parameters by Patient Cohort, Assessed Cycle 1 Day 1 and Cycle 2 Day 1

Parameter	DL1 Lenvatinib 20 mg + TMZ 100 mg/m² (*n* = 6)		DL2 Lenvatinib 24 mg + TMZ 100 mg/m² (*n* = 4)		DL3 Lenvatinib 24 mg + TMZ 150 mg/m² (*n* = 22)	
	Cycle 1/Day 1	Cycle 2/Day 1	Cycle 1/Day 1	Cycle 2/Day 1	Cycle 1/Day 1	Cycle 2/Day 1
t_{max} (h)	*n* = 6	*n* = 4	*n* = 4	*n* = 4	*n* = 22	*n* = 18
Mean (SD)	2.50 (1.30)	3.87 (3.28)	3.62 (1.70)	2.63 (0.48)	2.33 (1.44)	3.17 (1.65)
Median (min, max)	2.25(1.50, 4.98)	3.24(1.00, 8.00)	3.99(1.50, 5.00)	2.75(2.00, 3.00)	2.03(1.00, 7.98)	2.75(1.02, 8.00)
C_{max} (ng/mL)	*n* = 6	*n* = 4	*n* = 4	*n* = 4	*n* = 22	*n* = 18
Mean (SD)	498.5 (180.8)	498.0 (216.1)	407.0 (319.2)	430.8 (161.7)	571.2 (221.5)	573.9 (307.5)
Geometric mean (%CV)	467.73 (36.26)	460.4 (43.40)	327.8 (78.42)	411.0 (37.54)	524.3 (38.78)	491.2 (53.57)
$AUC_{(0\text{-}t)}$(ng·hr/mL)	*n* = 6	*n* = 4	*n* = 4	*n* = 4	*n* = 22	*n* = 18
Mean (SD)	3418.3 (1529.2)	4192.5 (815.0)	2642.5 (1304.5)	3027.5 (680.8)	3938.2 (1717.3)	4740.6 (2120.9)
Geometric mean (%CV)	3118.2 (44.74)	4123.4 (19.44)	2414.0 (49.37)	2970.6 (22.49)	3555.4 (43.61)	4284.5 (44.74)
$AUC_{(0\text{-}inf)}$ (ng·hr/mL)	*n* = 5	*n* = 2	*n* = 2	*n* = 4	*n* = 19	*n* = 13
Mean (SD)	3808.0 (1839.7)	4890.0 (113.1)	3375.0 (1803.1)	3440.0 (775.7)	4427.9 (1790.1)	5609.2 (2253.1)
Geometric mean (%CV)	3420.2 (48.31)	4889.4 (2.31)	3124.9 (53.43)	3375.7 (22.55)	4096.5 (40.43)	5216.6 (40.17)
$t_{1/2}$ (h)	*n* = 5	*n* = 2	*n* = 2	*n* = 4	*n* = 19	*n* = 13
Mean (SD)	5.86 (0.71)	5.55 (0.23)	5.43 (0.26)	5.45 (1.89)	6.14 (0.80)	6.57 (1.14)
Median (min, max)	5.69(5.18, 6.80)	5.55(5.39, 5.71)	5.43(5.25, 5.61)	5.86(2.88, 7.19)	6.07(4.60, 7.48)	6.32(5.21, 9.40)
CL/F (mL/h)	*n* = 5	*n* = 2	*n* = 2	*n* = 4	*n* = 19	*n* = 13
Mean (SD)	6610.0 (3862.4)	4090.0 (84.9)	8280.0 (4412.4)	6747.5 (2124.5)	6324.7 (2490.6)	4804.6 (1943.4)
Geometric mean (%CV)	5847.1 (58.43)	4089.6 (2.08)	7669.7 (53.29)	6489.7 (31.49)	5859.8 (39.38)	4471.2 (40.45)

$AUC_{0\text{-}inf}$, area under the plasma-concentration versus time curve from time zero to infinity; $AUC_{0\text{-}t}$, AUC from time zero to last quantifiable concentration; C_{max}, maximum plasma concentration; CL/F, oral clearance; %CV, coefficient of variation; DL, dose level; SD, standard deviation; t_{max}, time from dosing to maximum observed concentration; $t_{1/2}$, elimination half-life; TMZ, temozolomide.
